# *N*-[3-(2-Methyl­phen­yl)isoquinolin-1-yl]formamide

**DOI:** 10.1107/S1600536809011714

**Published:** 2009-04-02

**Authors:** Fu Na Cui, Jun Qi Li, Xiao Li Chen, Qing Bao Song

**Affiliations:** a The State Key Laboratory Breeding Base of Green Chemistry-Synthesis Technology, College of Chemical Engineering and Materials Science, Zhejiang University of Technology, Hangzhou 310014, People’s Republic of China

## Abstract

The title compound, C_17_H_14_N_2_O, crystallizes as a *cis* formamide isomer. The isoquinoline and benzene fragments are nearly perpendicular [dihedral angle = 81.79 (18)°], whereas the formamide group is virtually coplanar with the isoquinoline unit [dihedral angle = 1.66 (15)°]. Inter­molecular N—H⋯O hydrogen bonds link mol­ecules into a centrosymmetric dimer.

## Related literature

For the cytotoxic activity of aryl­isoquinolines, see: Cho *et al.* (2002 ▶, 2003 ▶). For the synthethic procedures relevant to this work, see: Nunno *et al.* (2008 ▶); Tovar & Swager (1999 ▶); Cho *et al.* (2002 ▶).
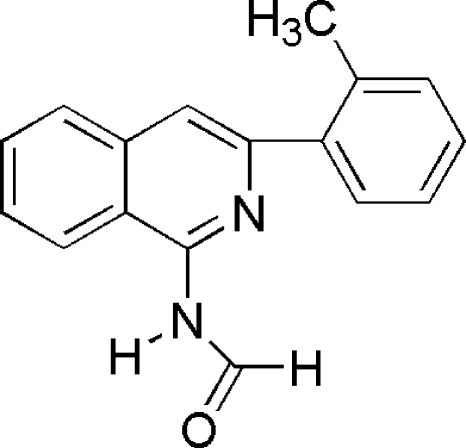


## Experimental

### 

#### Crystal data

C_17_H_14_N_2_O
*M* *_r_* = 262.30
Triclinic, 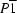

*a* = 5.3898 (14) Å
*b* = 11.166 (3) Å
*c* = 11.899 (3) Å
α = 106.139 (3)°
β = 93.128 (3)°
γ = 103.800 (3)°
*V* = 662.4 (3) Å^3^
*Z* = 2
Mo *K*α radiation
μ = 0.08 mm^−1^
*T* = 296 K
0.36 × 0.23 × 0.16 mm

#### Data collection

Bruker SMART CCD area-detector diffractometer
Absorption correction: multi-scan (*SADABS*; Bruker, 2000 ▶) *T*_min_ = 0.971, *T*_max_ = 0.987
4772 measured reflections
2399 independent reflections
1575 reflections with *I* > 2σ(*I*)
*R*_int_ = 0.017

#### Refinement

*R*[*F* ^2^ > 2σ(*F* ^2^)] = 0.043
*wR*(*F* ^2^) = 0.124
*S* = 1.03
2399 reflections
182 parameters
H-atom parameters constrained
Δρ_max_ = 0.13 e Å^−3^
Δρ_min_ = −0.19 e Å^−3^

### 

Data collection: *SMART* (Bruker, 2000 ▶); cell refinement: *SAINT* (Bruker, 2000 ▶); data reduction: *SAINT*; program(s) used to solve structure: *SHELXS97* (Sheldrick, 2008 ▶); program(s) used to refine structure: *SHELXL97* (Sheldrick, 2008 ▶); molecular graphics: *SHELXTL* (Sheldrick, 2008 ▶); software used to prepare material for publication: *SHELXTL*.

## Supplementary Material

Crystal structure: contains datablocks global, I. DOI: 10.1107/S1600536809011714/gk2201sup1.cif

Structure factors: contains datablocks I. DOI: 10.1107/S1600536809011714/gk2201Isup2.hkl

Additional supplementary materials:  crystallographic information; 3D view; checkCIF report

## Figures and Tables

**Table 1:** Hydrogen-bond geometry (Å, °)

*D*—H⋯*A*	*D*—H	H⋯*A*	*D*⋯*A*	*D*—H⋯*A*
N2—H2⋯O1^i^	0.86	2.10	2.940 (2)	165

Symmetry code: (i) 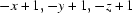.

**Fig. 1.**
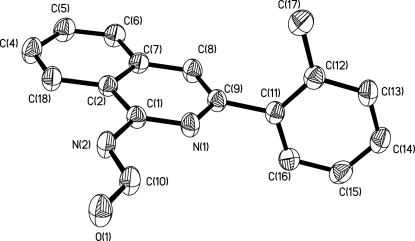
View of the molecular structure showing the atom-labelling scheme. Displacement ellipsoids are drawn at the 40% probability level. H atoms have been omitted for clarity.

**Fig. 2.**
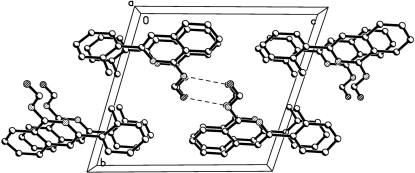
The molecular packing viewed along the a axis. Hydrogen bonds are shown with dashed lines. H atoms are omitted for clarity.

## supplementary crystallographic information

### Comment

Many of the arylisoquinoline derivatives exhibit potent cytotoxic activities against five different human tumor cell lines (Cho *et al.*, 2002, 2003). The title compound, that belongs to arylisoquinolines, has been synthesized to study its cytotoxic activity and its crystal structure is reported here.

### Experimental

A 2.5 *M* solution of n-BuLi in hexanes (54.5 mmol) was added to a solution of the diisopropylamine (59.9 mmol) in THF (5 ml) at 273 K under nitrogen atmosphere. After 10 min, the solution of 2-methylbenzonitrile (36.4 mmol) in THF (5 ml) was added dropwise and the obtained brown reaction mixture was stirred for 1 h, then adding the DMF (18.2 mmol), the mixture was stirred for 2 h at room temperature (Cho *et al.*, 2002; Nunno *et al.*, 2008; Tovar *et al.*, 1999). The mixture was subsequently concentrated under reduced pressure giving the crude product. The residue was recrystallized from ethanol. Colorless crystals of the title compound were obtained by slow evaporation of the solvent after 2 days at room temperature(Yield: 73%, m.p. 401–403 K).

### Refinement

All H atoms were placed in calculated posistion with C—H = 0.93 - 0.96 Å, and N—H = 0.86Å and refined in the riding mode aproximation with *U*_iso_(H) = 1.2*U*_eq_ of the carrier atom.

### Crystal data

**Table:**

C_17_H_14_N_2_O	*Z* = 2
*M**_r_* = 262.30	*F*(000) = 276
Triclinic, *P*1	*D*_x_ = 1.315 Mg m^−^^3^
Hall symbol: -P 1	Mo *K*α radiation, λ = 0.71073 Å
*a* = 5.3898 (14) Å	Cell parameters from 1222 reflections
*b* = 11.166 (3) Å	θ = 3.0–26.0°
*c* = 11.899 (3) Å	µ = 0.08 mm^−^^1^
α = 106.139 (3)°	*T* = 296 K
β = 93.128 (3)°	Block, colourless
γ = 103.800 (3)°	0.36 × 0.23 × 0.16 mm
*V* = 662.4 (3) Å^3^	

### Data collection

**Table:**

Bruker SMART CCD area-detector diffractometer	2399 independent reflections
Radiation source: fine-focus sealed tube	1575 reflections with *I* > 2σ(*I*)
graphite	*R*_int_ = 0.017
Detector resolution: 0 pixels mm^-1^	θ_max_ = 25.5°, θ_min_ = 3.0°
φ and ω scans	*h* = −6→6
Absorption correction: multi-scan (*SADABS*; Bruker, 2000)	*k* = −13→13
*T*_min_ = 0.971, *T*_max_ = 0.987	*l* = −14→14
4772 measured reflections	

### Refinement

**Table:**

Refinement on *F*^2^	Primary atom site location: structure-invariant direct methods
Least-squares matrix: full	Secondary atom site location: difference Fourier map
*R*[*F*^2^ > 2σ(*F*^2^)] = 0.043	Hydrogen site location: inferred from neighbouring sites
*wR*(*F*^2^) = 0.124	H-atom parameters constrained
*S* = 1.03	*w* = 1/[σ^2^(*F*_o_^2^) + (0.0578*P*)^2^ + 0.0805*P*] where *P* = (*F*_o_^2^ + 2*F*_c_^2^)/3
2399 reflections	(Δ/σ)_max_ < 0.001
182 parameters	Δρ_max_ = 0.13 e Å^−^^3^
0 restraints	Δρ_min_ = −0.19 e Å^−^^3^

### Special details

**Table:**

Geometry. All e.s.d.'s (except the e.s.d. in the dihedral angle between two l.s. planes)are estimated using the full covariance matrix. The cell e.s.d.'s are takeninto account individually in the estimation of e.s.d.'s in distances, anglesand torsion angles; correlations between e.s.d.'s in cell parameters are onlyused when they are defined by crystal symmetry. An approximate (isotropic)treatment of cell e.s.d.'s is used for estimating e.s.d.'s involving l.s. planes.
Refinement. Refinement of *F*^2^ against ALL reflections. The weighted *R*-factor *wR* and goodness of fit *S* are based on *F*^2^, conventional *R*-factors *R* are based on *F*, with *F* set to zero for negative *F*^2^. The threshold expression of *F*^2^ > σ(*F*^2^) is used only for calculating *R*-factors(gt) *etc*. and is not relevant to the choice of reflections for refinement. *R*-factors based on *F*^2^ are statistically about twice as large as those based on *F*, and *R*- factors based on ALL data will be even larger.

### Fractional atomic coordinates and isotropic or equivalent isotropic displacement parameters (Å^2^)

**Table:**

	*x*	*y*	*z*	*U*_iso_*/*U*_eq_	
C1	0.2507 (3)	0.67650 (17)	0.71903 (14)	0.0446 (4)	
C2	0.1010 (3)	0.74496 (17)	0.67117 (15)	0.0444 (4)	
C4	−0.0799 (4)	0.8081 (2)	0.51425 (18)	0.0651 (6)	
H4	−0.1015	0.8032	0.4349	0.078*	
C5	−0.1976 (4)	0.8866 (2)	0.59439 (18)	0.0627 (6)	
H5	−0.2959	0.9339	0.5681	0.075*	
C6	−0.1697 (4)	0.8945 (2)	0.71022 (18)	0.0592 (5)	
H6	−0.2504	0.9465	0.7627	0.071*	
C7	−0.0188 (3)	0.82420 (18)	0.75214 (15)	0.0481 (5)	
C8	0.0201 (4)	0.83123 (19)	0.87248 (16)	0.0550 (5)	
H8	−0.0586	0.8820	0.9272	0.066*	
C9	0.1717 (4)	0.76428 (18)	0.90878 (15)	0.0485 (5)	
C10	0.5217 (4)	0.52789 (19)	0.67853 (16)	0.0551 (5)	
H10	0.5431	0.5346	0.7583	0.066*	
C11	0.2307 (4)	0.77304 (19)	1.03607 (15)	0.0479 (5)	
C12	0.0620 (4)	0.69894 (19)	1.09041 (16)	0.0531 (5)	
C13	0.1360 (4)	0.7069 (2)	1.20679 (17)	0.0623 (6)	
H13	0.0261	0.6567	1.2437	0.075*	
C14	0.3655 (4)	0.7863 (2)	1.26835 (18)	0.0636 (6)	
H14	0.4105	0.7889	1.3457	0.076*	
C15	0.5290 (4)	0.8620 (2)	1.21612 (18)	0.0662 (6)	
H15	0.6831	0.9178	1.2584	0.079*	
C16	0.4630 (4)	0.8548 (2)	1.09957 (17)	0.0600 (6)	
H16	0.5751	0.9051	1.0635	0.072*	
C17	−0.1907 (4)	0.6097 (2)	1.0258 (2)	0.0735 (6)	
H17A	−0.3010	0.6596	1.0076	0.110*	
H17B	−0.2710	0.5602	1.0746	0.110*	
H17C	−0.1617	0.5524	0.9541	0.110*	
C18	0.0666 (4)	0.7385 (2)	0.55094 (16)	0.0558 (5)	
H18	0.1441	0.6866	0.4966	0.067*	
N1	0.2872 (3)	0.68582 (15)	0.83158 (12)	0.0492 (4)	
N2	0.3716 (3)	0.59354 (15)	0.64362 (12)	0.0517 (4)	
H2	0.3469	0.5844	0.5694	0.062*	
O1	0.6325 (3)	0.45974 (14)	0.61283 (11)	0.0655 (4)	

### Atomic displacement parameters (Å^2^)

**Table:**

	*U*^11^	*U*^22^	*U*^33^	*U*^12^	*U*^13^	*U*^23^
C1	0.0521 (11)	0.0470 (11)	0.0386 (10)	0.0214 (9)	0.0112 (8)	0.0112 (8)
C2	0.0477 (10)	0.0478 (11)	0.0410 (10)	0.0187 (9)	0.0078 (8)	0.0134 (8)
C4	0.0777 (15)	0.0831 (16)	0.0477 (11)	0.0425 (13)	0.0049 (10)	0.0231 (11)
C5	0.0683 (13)	0.0731 (15)	0.0596 (12)	0.0387 (12)	0.0033 (10)	0.0241 (11)
C6	0.0639 (13)	0.0663 (14)	0.0569 (12)	0.0364 (11)	0.0094 (10)	0.0168 (10)
C7	0.0492 (11)	0.0519 (12)	0.0466 (10)	0.0206 (9)	0.0068 (8)	0.0142 (9)
C8	0.0668 (13)	0.0630 (13)	0.0426 (10)	0.0340 (11)	0.0147 (9)	0.0119 (9)
C9	0.0555 (11)	0.0555 (12)	0.0384 (10)	0.0238 (10)	0.0114 (8)	0.0116 (9)
C10	0.0755 (14)	0.0638 (13)	0.0380 (10)	0.0378 (12)	0.0107 (9)	0.0170 (9)
C11	0.0579 (12)	0.0535 (11)	0.0383 (9)	0.0285 (10)	0.0111 (9)	0.0104 (9)
C12	0.0595 (12)	0.0560 (12)	0.0463 (11)	0.0227 (10)	0.0070 (9)	0.0132 (9)
C13	0.0754 (15)	0.0728 (15)	0.0434 (11)	0.0228 (12)	0.0084 (10)	0.0220 (10)
C14	0.0760 (15)	0.0762 (15)	0.0417 (11)	0.0293 (13)	0.0017 (11)	0.0157 (11)
C15	0.0628 (13)	0.0766 (15)	0.0529 (12)	0.0210 (12)	−0.0034 (10)	0.0094 (11)
C16	0.0576 (13)	0.0731 (14)	0.0484 (11)	0.0181 (11)	0.0083 (10)	0.0159 (10)
C17	0.0690 (14)	0.0802 (16)	0.0671 (14)	0.0124 (13)	−0.0022 (11)	0.0239 (12)
C18	0.0651 (13)	0.0687 (14)	0.0419 (10)	0.0332 (11)	0.0089 (9)	0.0163 (10)
N1	0.0622 (10)	0.0564 (10)	0.0363 (8)	0.0285 (8)	0.0113 (7)	0.0140 (7)
N2	0.0707 (11)	0.0640 (10)	0.0326 (8)	0.0390 (9)	0.0089 (7)	0.0153 (7)
O1	0.0930 (11)	0.0775 (10)	0.0454 (7)	0.0555 (9)	0.0183 (7)	0.0194 (7)

### Geometric parameters (Å, °)

**Table:**

C1—N1	1.314 (2)	C10—N2	1.334 (2)
C1—N2	1.406 (2)	C10—H10	0.9300
C1—C2	1.430 (2)	C11—C12	1.390 (3)
C2—C18	1.412 (2)	C11—C16	1.393 (3)
C2—C7	1.412 (2)	C12—C13	1.393 (3)
C4—C18	1.365 (3)	C12—C17	1.500 (3)
C4—C5	1.395 (3)	C13—C14	1.367 (3)
C4—H4	0.9300	C13—H13	0.9300
C5—C6	1.354 (3)	C14—C15	1.368 (3)
C5—H5	0.9300	C14—H14	0.9300
C6—C7	1.416 (3)	C15—C16	1.388 (3)
C6—H6	0.9300	C15—H15	0.9300
C7—C8	1.413 (2)	C16—H16	0.9300
C8—C9	1.358 (3)	C17—H17A	0.9600
C8—H8	0.9300	C17—H17B	0.9600
C9—N1	1.369 (2)	C17—H17C	0.9600
C9—C11	1.502 (2)	C18—H18	0.9300
C10—O1	1.218 (2)	N2—H2	0.8600
			
N1—C1—N2	116.00 (15)	C16—C11—C9	118.94 (17)
N1—C1—C2	124.34 (16)	C11—C12—C13	118.20 (19)
N2—C1—C2	119.66 (15)	C11—C12—C17	121.62 (17)
C18—C2—C7	118.91 (16)	C13—C12—C17	120.16 (19)
C18—C2—C1	124.80 (16)	C14—C13—C12	121.9 (2)
C7—C2—C1	116.28 (15)	C14—C13—H13	119.0
C18—C4—C5	120.80 (19)	C12—C13—H13	119.0
C18—C4—H4	119.6	C13—C14—C15	120.03 (19)
C5—C4—H4	119.6	C13—C14—H14	120.0
C6—C5—C4	120.51 (18)	C15—C14—H14	120.0
C6—C5—H5	119.7	C14—C15—C16	119.5 (2)
C4—C5—H5	119.7	C14—C15—H15	120.2
C5—C6—C7	120.56 (18)	C16—C15—H15	120.2
C5—C6—H6	119.7	C15—C16—C11	120.7 (2)
C7—C6—H6	119.7	C15—C16—H16	119.7
C2—C7—C8	118.35 (16)	C11—C16—H16	119.7
C2—C7—C6	119.00 (16)	C12—C17—H17A	109.5
C8—C7—C6	122.64 (17)	C12—C17—H17B	109.5
C9—C8—C7	120.43 (17)	H17A—C17—H17B	109.5
C9—C8—H8	119.8	C12—C17—H17C	109.5
C7—C8—H8	119.8	H17A—C17—H17C	109.5
C8—C9—N1	122.13 (16)	H17B—C17—H17C	109.5
C8—C9—C11	122.85 (16)	C4—C18—C2	120.22 (18)
N1—C9—C11	115.00 (15)	C4—C18—H18	119.9
O1—C10—N2	124.36 (17)	C2—C18—H18	119.9
O1—C10—H10	117.8	C1—N1—C9	118.43 (15)
N2—C10—H10	117.8	C10—N2—C1	124.96 (15)
C12—C11—C16	119.63 (17)	C10—N2—H2	117.5
C12—C11—C9	121.41 (17)	C1—N2—H2	117.5
			
N1—C1—C2—C18	178.11 (18)	C9—C11—C12—C13	176.52 (17)
N2—C1—C2—C18	−1.4 (3)	C16—C11—C12—C17	179.84 (18)
N1—C1—C2—C7	−1.8 (3)	C9—C11—C12—C17	−1.9 (3)
N2—C1—C2—C7	178.68 (16)	C11—C12—C13—C14	1.1 (3)
C18—C4—C5—C6	−0.4 (3)	C17—C12—C13—C14	179.6 (2)
C4—C5—C6—C7	0.6 (3)	C12—C13—C14—C15	0.6 (3)
C18—C2—C7—C8	−179.13 (18)	C13—C14—C15—C16	−1.7 (3)
C1—C2—C7—C8	0.8 (3)	C14—C15—C16—C11	1.0 (3)
C18—C2—C7—C6	0.1 (3)	C12—C11—C16—C15	0.7 (3)
C1—C2—C7—C6	−179.99 (17)	C9—C11—C16—C15	−177.58 (18)
C5—C6—C7—C2	−0.4 (3)	C5—C4—C18—C2	0.0 (3)
C5—C6—C7—C8	178.7 (2)	C7—C2—C18—C4	0.1 (3)
C2—C7—C8—C9	0.8 (3)	C1—C2—C18—C4	−179.81 (19)
C6—C7—C8—C9	−178.41 (19)	N2—C1—N1—C9	−179.35 (16)
C7—C8—C9—N1	−1.5 (3)	C2—C1—N1—C9	1.1 (3)
C7—C8—C9—C11	176.90 (18)	C8—C9—N1—C1	0.6 (3)
C8—C9—C11—C12	83.5 (3)	C11—C9—N1—C1	−177.95 (17)
N1—C9—C11—C12	−98.0 (2)	O1—C10—N2—C1	−177.35 (19)
C8—C9—C11—C16	−98.3 (2)	N1—C1—N2—C10	−1.6 (3)
N1—C9—C11—C16	80.3 (2)	C2—C1—N2—C10	177.92 (17)
C16—C11—C12—C13	−1.7 (3)		

### Hydrogen-bond geometry (Å, °)

**Table:**

*D*—H···*A*	*D*—H	H···*A*	*D*···*A*	*D*—H···*A*
N2—H2···O1^i^	0.86	2.10	2.940 (2)	165

Symmetry codes: (i) −*x*+1, −*y*+1, −*z*+1.
